# A Doubtful Witness

**Published:** 1854-07

**Authors:** 


### A DOUBTFUL WITNESS.

Professional engagements required the writer's presence in a circuit court which was then in session in one of the villages of a midland county of the "Empire State;" and during the term an incident occurred, which may be interesting, if not useful to those legal gentlemen who are partial to the study of the "laws of evidence." The case tried was one in which a question arose as to personal property, claimed to have been sold some time previously under an execution, and the plaintiff in the case called a witness to establish the fact of the sale. The following "evidence" was elicited on the cross-examination of the witness:—

*Question by Counsel.* "Sir, you say you attended the sale on the execution spoken of. Did you keep the minutes of that sale?"

*Witness.* "Don't know, sir, but I did; don't recollect whether I kept the minutes, or the sheriff, or nobody, I think it was one of us."

*Counsel.* "Well, sir, will you tell me what articles were sold at the execution?"

[Here the witness hesitated, not willing to commit himself by going into particulars, until the patience of the counsel became exhausted, and he pressed a special interrogatory.]

*Counsel.* "Did you on that occasion sell a threshing-machine?"

*Witness.* "Yes, I think we did."

*Counsel.* "I wish you to be positive. Are you *sure* of it?"

*Witness.* "Can't say I am sure of it; and when I come to think of it, I don't know as we did; think we didn't."

*Counsel.* "Will you swear, then, that you did not sell one?"

*Witness.* "No, sir; don't think I would; for I can't say whether we did or didn't."

*Counsel* "Did you sell a horse-power?"

*Witness.* "Horse-power?"

*Counsel.* "Yes, horse-power?"

*Witness.* "Horse-power! Well, it seems to me we did. And it seems to me we didn't. I don't know as I can recollect whether I remember there was any horse-power there; and if there wasn't any there, I can't say whether we sold it or not, but

I don't *think* we did; though it may be, perhaps, that we *did*, after all. It's some time ago, and I don't like to say certainly."

*Counsel.* "Well, perhaps you can tell me this; did you sell a fanning-mill?"

*Witness.* "Yes, sir, we sold a fanning-mill. I guess I am sure of that."

*Counsel.* "Well, you swear to that, do you?—that one thing, though I don't see it on the list."

*Witness.* "Why, I may be mistaken about it; perhaps I am. It may be it was somebody else's fanning-mill at some other time; not sure."

*Counsel, (to the Court.)* "I should like to know, may it please the Court, what this witness does know, and what he is sure of."

*Witness, (to Counsel.)* "Well, sir, I know one thing that I'm sure of; and that is, that on that sale we sold either a *threshing-machine,* or a *horse-power,* or a *fanning-mill,* or one, or all, or neither of them, but don't know which!"

